# Releasing Intracellular NS1 from Mosquito Cells for the Detection of Dengue Virus-Infected Mosquitoes

**DOI:** 10.3390/v12101105

**Published:** 2020-09-29

**Authors:** Lie Cheng, Wei-Liang Liu, Hsing-Han Li, Matthew P. Su, Shih-Cheng Wu, Hsin-Wei Chen, Chao-Ying Pan, Jih-Jin Tsai, Chun-Hong Chen

**Affiliations:** 1 National Institute of Infectious Diseases and Vaccinology, National Health Research Institutes, Miaoli 350401, Taiwan; milkacheng@gmail.com (L.C.); ligigi1026@gmail.com (H.-H.L.); chenhw@nhri.org.tw (H.-W.C.); 2 Tropical Medicine Center, Kaohsiung Medical University Hospital, Kaohsiung 807377, Taiwan; 3 National Mosquito-Borne Diseases Control Research Center, National Health Research Institutes, Miaoli 350401, Taiwan; weiliangliu58@gmail.com (W.-L.L.); scwu@nhri.edu.tw (S.-C.W.); 4 Institution of Biotechnology, National Tsing Hua University, Hsinchu 300044, Taiwan; 5 Department of Biological Science, Nagoya University, Nagoya 464-8601, Japan; su.matthew.paul@h.mbox.nagoya-u.ac.jp; 6 Graduate Institute of Biomedical Sciences, China Medical University, Taichung 110001, Taiwan; 7 Graduate Institute of Medicine, College of Medicine, Kaohsiung Medical University, Kaohsiung 807378, Taiwan; 8 Department of Health, Kaohsiung City Government, Kaohsiung 800852, Taiwan; panjoe2000@gmail.com; 9 Division of Infectious Diseases, Department of Internal Medicine, Kaohsiung Medical University Hospital, Kaohsiung 807377, Taiwan; 10 School of Medicine, College of Medicine, Kaohsiung Medical University, Kaohsiung 807378, Taiwan

**Keywords:** dengue virus, flavivirus, NS1, NS1 secretion, mosquito surveillance, rapid test

## Abstract

Dengue virus (DENV), the pathogen that causes dengue fever, is mainly transmitted by *Aedes aegypti*. Surveillance of infected mosquitoes is a major component of integrated mosquito control methods for reducing the risk of vector-born disease outbreaks. However, a specialized rapid test for DENV detection in mosquitoes is not currently available. Utilizing immunoblotting, we found that the secretion of NS1 from both a DENV-infected mosquito cell line and mosquito bodies was below the detection threshold. However, when Triton X-100 was used to lyse infected mosquitoes, intracellular NS1 was released, and could then be effectively detected by the NS1 rapid test. The distribution of DENV NS1 in intrathoracically infected mosquitoes was different from that of orally infected mosquitoes. Next, we performed sensitivity tests by bisecting mosquitoes longitudinally; one half of each mosquito was subjected to the NS1 rapid test while the other half was used for qPCR confirmation. This modified test had a sensitivity of nearly 90% from five days post-infection onwards, while DENV had escaped from the midgut barrier. This adapted test offers a valuable, easy-to-use tool for mosquito surveillance, which is a crucial component of DENV disease control.

## 1. Introduction

Dengue virus (DENV) is a flavivirus with four distinct serotypes (DENV 1–4), and is predominantly transmitted by *Aedes aegypti* (*A. aegypti*) and *Aedes albopictus*. Infection with DENV can cause dengue fever, dengue hemorrhagic fever and dengue shock syndrome [1,2]. Over 2 billion people worldwide are at risk of dengue infection, with around 390 million cases documented each year [3]. There are no currently available therapies for dengue infection, and the most advanced vaccine is limited in applicability [4]. Alternative methods of DENV control are therefore necessary, and the majority of these are focused on vector management [5].

The proactive surveillance of mosquito density and DENV infection rates in high-risk areas is one strategy for vector control [6]. By identifying changes in mosquito population size or the viral infection rate within the mosquito population, targeted interventions can be made; this reduces associated costs as well as the likelihood of disease outbreaks [7]. Mosquito surveillance requires sensitive tests to detect the viral infection status of mosquitoes caught in traps [8]. However, while several methods exist for detecting dengue infection in humans, such as virus isolation, ELISA, qPCR and the dengue non-structural protein 1 (NS1) rapid test [9,10,11,12,13], there are currently no readily available and specialized rapid tests for detecting DENV in mosquitoes. Adapting one of these tests for use in mosquitoes could therefore be of great value for mosquito control programs.

The dengue NS1 antigen (Ag) rapid test is a solid-phase immunochromatographic (ICT) test, also known as a “one-step dengue test”, for the rapid, qualitative detection of DENV NS1 in human serum, plasma, or whole blood. It has not previously been used outside of a research context to test mosquitoes. Currently, the NS1 rapid test is a cost effective, time-saving and easy-to-use method for identifying DENV-infected patients. The NS1 rapid test therefore has significant potential for testing infected mosquitoes if it can be adapted for use in invertebrates.

In humans, NS1 can be used as an indicator of DENV infection because it is released into the blood and is positively correlated with virus replication [14]. In vertebrate cells, NS1 is secreted efficiently into the extracellular space and correlates with the modulation of the immune response (e.g., inhibition of complement system function [15,16] and increased vascular leakage [17]). However, the secretion of NS1 by invertebrate cells remains controversial [16,18,19].

In this study, we found that the secretion of DENV NS1 was limited in infected mosquito cell lines, although intracellular NS1 expression was similar in both mammalian and mosquito cell lines. The application of detergent greatly facilitated the utilization of the rapid test for detecting DENV NS1 in orally infected mosquitoes. We found that detecting DENV infection in mosquitoes that had been infected via thorax injection, however, did not require detergent. We therefore modified the gridding buffer used as part of the commercial NS1 rapid test strip. We next investigated the efficiency of DENV detection in infected mosquitoes by comparing the results of this improved method with qPCR data collected from each mosquito. We found that our modified rapid test successfully identified 84–89% of infected mosquitoes 5–21 days post-infection (PI). Even as early as three days PI, almost 60% of infected mosquitoes could be identified. This adapted NS1 rapid test could therefore be an important tool for use in DENV management, helping to reduce the risk of DENV infection.

## 2. Materials and Methods

### 2.1. Mosquito, Cell Culture and Virus Maintenance

The Higgs strain of *A. aegypti* was used throughout this study. Adult mosquitoes were kept at 28 °C and 75% relative humidity in 12:12 h light:dark conditions and provided with a constant supply of 10% sucrose water. Vero and Baby Hamster Kidney (BHK) cells were maintained in Dulbecco’s modified Eagle medium (DMEM) with 10% fetal bovine serum, 100 U/mL penicillin and 100 μg/mL streptomycin at 37 °C. C6/36 cell were maintained in Roswell Park Memorial Institute (RPMI) 1640 with 10% fetal bovine serum, 100 U/mL penicillin and 100 μg/mL streptomycin at 28 °C. DENV1 (CDC2014 strain), DENV2 (NGC strain), DENV3 (DN8700829A strain) and DENV4 (DN8000475A strain) were produced using the C6/36 (DENV1) or Vero (DENV2, DENV3, and DENV4) cell line and stored in a −80 °C freezer. Virus titers were determined via plaque assays using BHK cells.

### 2.2. Plaque Assays

To determine the DENV2 titer, 2 × 10^5^ BHK cells were seeded into each well of a 6-well plate and incubated for 24 h. Virus supernatant was serially diluted (from 10^−2^ to 10^−6^) with DMEM. Diluted virus was then used to infect the BHK cells in the 6-well plate, which was then kept in a cell culture incubator for 2 h before the supernatant was removed. After a single phosphate-buffered saline (PBS) wash, the cells were covered with DMEM containing 1% methyl cellulose and 2% fetal bovine serum and incubated for a further five days. Post incubation, the DMEM was removed and the cells were fixed and stained with cell staining solution (0.5% crystal violet, 1.85% formaldehyde, 50% ethanol, and 0.85% NaCl) for 2 h, followed by washing out excess cell staining solution with H_2_O. All the samples were tested in triplicate. Plaques were calculated as plaque-forming units (PFU).

### 2.3. Infecting Cells or Mosquitoes with DENV

To infect Vero and C6/36 cells, 6 × 10^5^ cells were seeded into a 60 mm dish. After overnight incubation, cells were infected with DENV (multiplicity of infection (MOI) = 0.5) in serum free DMEM for 2 h at 37 °C. Infected cells were then washed with PBS and we added DMEM with 10% fetal bovine serum. We then incubated these cells for another 48 h at 37 °C for the immunoblotting analysis.

To infect mosquitoes orally with DENV, fresh mouse blood (BALB/c Mice; BioLASCO Taiwan Co., Ltd., Taipei, Taiwan) was centrifuged at 4 °C for 10 min to separate plasma and blood cells. The plasma component was then heat treated at 56 °C for 1 h. Blood cells were washed three times with PBS. All of the treated plasma and blood cells were then combined. DENV virus supernatant was mixed with the treated mouse blood to create a 10^7^ PFU/mL virus–blood mixture. This mixture was fed to mosquitoes via an artificial membrane feeder for 30 min. Successfully blood-fed mosquitoes were then maintained as normal.

To infect mosquitoes with DENV via thoracic injections, mosquitoes were first divided into four groups (25 mosquitoes for each serotype). A quantity of 400 PFU of one of the DENV serotypes was then injected into the mosquito thorax using a micro-injector (Nanoject II, Drummond Scientific Company, Broomall, PA, USA). Virus-injected mosquitoes were then maintained as normal.

### 2.4. Immunoblotting

NS1 levels from infected cells or mosquitoes were investigated using immunoblotting. For cell line analysis, the supernatant of incubated cells was harvested followed by centrifugation at 1000× *g* for 5 min at 4 °C to remove cell debris. Cells were washed with PBS twice and then lysed with RIPA lysis buffer. Lysates were centrifuged at 15,000× *g* for 5 min at 4 °C, before supernatants were then transferred to new eppendorfs.

For mosquito samples, three whole mosquito bodies were homogenized with RIPA lysis buffer. Lysates were then centrifuged at 15,000× *g* for 5 min at 4 °C, before supernatants were then transferred to new eppendorfs.

For immunoblotting, 20 μL of treated culture supernatants and protein samples (10 μg) from cell lines or mosquito lysates were resolved by 10% SDS-PAGE. Proteins were then transferred to PVDF membrane (Merck, Darmstadt, Germany). Blots were blocked with 3% skimmed milk in TBST (Tris-Buffered Saline-0.1% Tween 20) buffer for 1 h at room temperature. Primary antibodies, rabbit polyclonal NS1 (Cat# PA5-32207; Invitrogen, Waltham, MA, USA) or rabbit polyclonal beta-actin (Genetex, Alton Pkwy Irvine, CA, USA) in blocking buffer were applied to blots and incubated for 1 h at room temperature. Blots were then incubated with HRP conjugated anti-rabbit secondary antibody for 1 h at room temperature. Before and after secondary antibody incubation, blots were washed with TBST three times. After washing, blots were incubated with Immobilon Western Chemiluminescent HRP Substrate (Millipore, Darmstadt, Germany) and signals were detected using an Amersham imager 600 (GE Healthcare, Chicago, IL, USA).

### 2.5. Dengue NS1 Rapid Test

Mosquito infection status was tested at an individual level. Mosquitoes were harvested at specific post-infection time points and bisected longitudinally. One half of each mosquito was homogenized with either PBS or 0.1%, 0.5% or 1% Triton X-100-PBS. The liquid component of the treated lysate was subjected to the SD BioLine Dengue NS1 rapid test (11FK50; Standard Diagnostics, Seoul, Korea).

### 2.6. RNA Extraction, Reverse Transcription and qPCR

The other half of each mosquito was used for qPCR confirmation of infection status. Prior to qPCR analysis, the sample was homogenized with TRI reagent (Merck, Darmstadt, Germany) and RNA was extracted following the manufacturer’s protocol. Extracted total RNA was immediately reverse transcribed using SuperScript III Reverse Transcriptase and Random Primers (Thermo Fisher Scientific, Waltham, MA, USA). A quantity of 2 μg total RNA was treated with amplification grade DNase I (Thermo Fisher Scientific), followed by the first-strand cDNA synthesis step. A quantity of cDNA equivalent to 10 ng total RNA was used as a sample for the real-time PCR analysis. Real-time PCR was performed using KAPA SYBR FAST ROX Low (KAPA Biosystems, Darmstadt, Germany) on a ViiA 7 real-time PCR system (Thermo Fisher Scientific). A Ct value > 35 cycles was considered to be negative.

### 2.7. Immunofluorescence Staining and Imaging

Midgut, fat, muscle and salivary glands were dissected from mosquitoes at three, five or seven days post DENV infection. The harvested tissues were fixed and permeabilized using a fixative buffer (4% paraformaldehyde, 0.1 M HEPES and 1% Triton X-100 in PBS) for 30 min. Samples were then washed four times with 0.1% Triton X-100-PBS, before being blocked with 3% BSA for 2 h at room temperature.

Blocked tissues were stained with rabbit polyclonal NS1 (Cat# PA5-32207; Invitrogen, Waltham, MA, USA) and mouse monoclonal E protein (Cat# GTX629116; Genetex, Alton Pkwy Irvine, CA, USA) primary antibodies at 4 °C overnight, followed by incubation with anti-Ms-Alexa-488 and anti-Rb-Alexa-594 (Invitrogen) secondary antibodies for 3 h at room temperature. Before and after secondary antibody staining, tissues were washed four times with 0.1% Triton X-100-PBS. Tissues were then stained with DAPI for 8 min and washed with PBS. After mounting with mounting gel, tissues were imaged using a Leica TCS SP5 confocal microscope (Leica, Wetzlar, Germany).

### 2.8. Statistical Analysis

All analyses were conducted using the R software package [20]. Sensitivity was calculated as

$$\frac{number\;of\;positive\;rapid\;tests}{number\;of\;positive\;qPCR\;tests}$$

Specificity was calculated as

$$\frac{number\;of\;negative\;rapid\;tests}{number\;of\;negative\;qPCR\;tests}$$

Cohen’s kappa was used to evaluate levels of agreement between the results of rapid tests and qPCR [21]. Kappa values between 0.41 and 0.60 indicate moderate agreement, 0.61–0.80 indicate substantial agreement, and 0.81–0.99 indicate almost perfect agreement.

## 3. Results

### 3.1. DENV NS1 Is not Secreted at Detectable Levels by Mosquito Cell Lines

If the secreted form of NS1 can be found in mosquito body fluids, the detection of mosquito infection status using the NS1 rapid test may be possible in a manner similar to that achieved via the testing of human blood or saliva.

**Figure 1:**
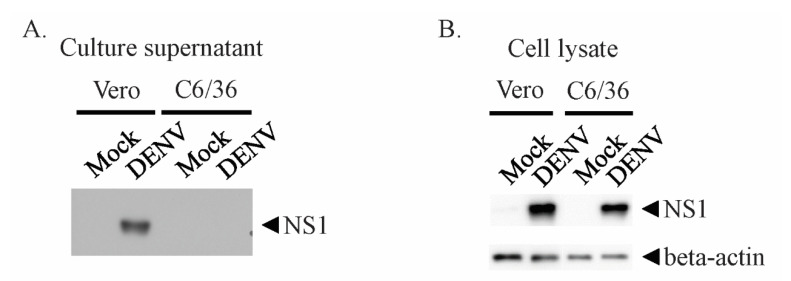
NS1 is secreted at undetectable levels in mosquito cell lines. Vero and C6/36 cells were infected with dengue virus (DENV) 2 at multiplicity of infection (MOI) = 0.5. After two days of incubation, culture supernatants and cells were harvested. The cells were lysed with 1% Triton X-100-phosphate-buffered saline (PBS) buffer. (**A**) Cell culture supernatant (20 µL) and (**B**) cell lysate (10 µg) were subjected to immunoblotting with non-structural protein 1 (NS1) antibody.

To test the secretion levels of NS1 from mammalian and mosquito cells, we parallelly infected Vero and C6/36 cells with DENV2. We detected NS1 in the supernatant from infected Vero cells only (Figure 1A). However, when the cells were lysed, similar levels of NS1 were found in both cell lines (Figure 1B). These results suggested that the NS1 protein was predominantly presenting within mosquito cells, and that secretion from the cell was at undetectable levels.

### 3.2. Detergent Releases Intracellular NS1 from Infected Mosquitoes for Rapid Test Detection

**Figure 2:**
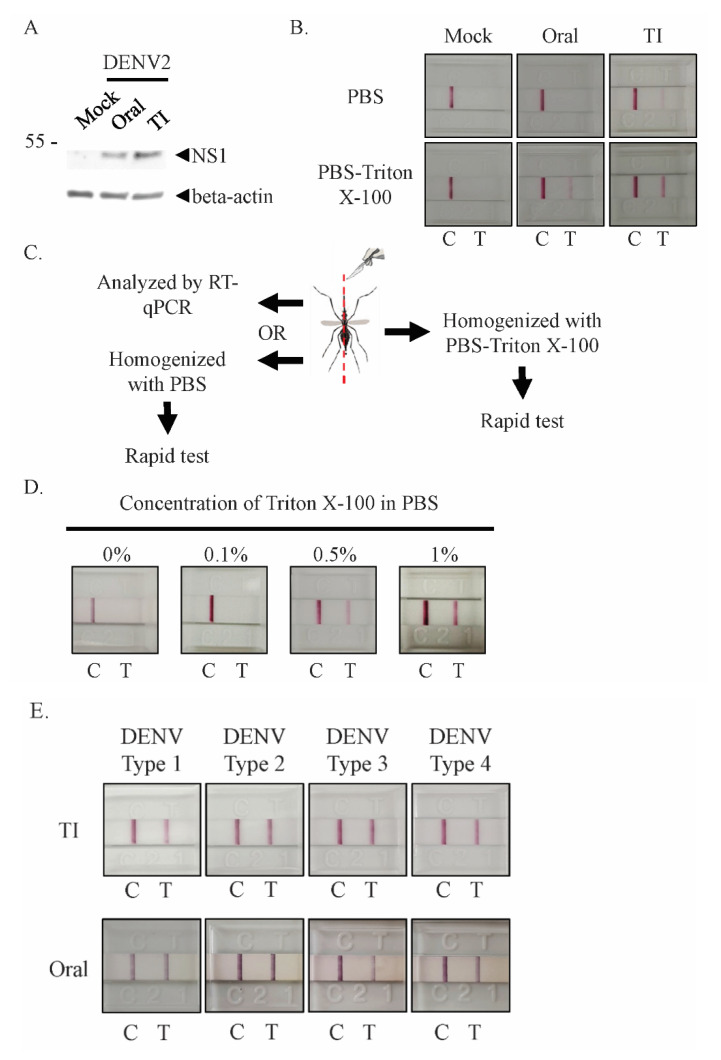
NS1 can be detected in lysate from mosquito bodies when Triton X-100-PBS lysis buffer is used. (**A**) Mosquitoes were infected with DENV2 orally (oral) or via thorax injection (TI). After seven days of incubation, infected mosquitoes were homogenized with lysis buffer. Soluble lysates were separated with SDS-PAGE and immunoblotting was performed with anti-NS1 and beta-actin antibodies. (**B**–**E**) Mosquitoes were orally (oral) or intrathoracically infected (TI) with DENV2 and incubated for seven days. (**B**) Incubated mosquitoes were homogenized with PBS or 1% Triton X-100-PBS buffer; both lysates were then tested using the NS1 rapid test. (**C**) To confirm the infection status of orally infected mosquitoes, mosquitoes were longitudinally and symmetrically bisected for homogenization in different lysis buffers or for analysis with different methods. (**D**) The Triton X-100-PBS lysis buffer dose-dependent assay for orally infected mosquitoes. Half of each infected mosquito was homogenized with 0.1–1% Triton X-100-PBS buffer and tested using the NS1 rapid test strip. (**E**) Mosquitoes were infected with one of the four DENV serotypes via thorax injection or oral infection, before being homogenized with 1% Triton X-100-PBS and tested using the rapid test strip. At least 5 individual mosquitoes were tested per sample. The DENV titer from serotype1 to 4 of TI infected mosquito was 2.2 × 10^4^, 4.0 × 10^4^, 2.9 × 10^4^, and 2.3 × 10^4^, respectively. The virus titer from serotype1 to 4 of orally infected mosquito was 8.9 × 10^3^, 1.1 × 10^4^, 6.8 × 10^3^, and 8.0 × 10^3^, respectively. All control (C) and positive (T) signals were observed within 20 min.

Next, to test whether NS1 could be detected in the whole bodies of infected mosquitoes, we infected mosquitoes with DENV2 via either oral exposure or thorax injections. Infection via the oral route is the natural method by which mosquitoes become infected with DENV, but direct injection ensures infection rates (number of mosquitoes infected with DENV normalized by the total number injected) of almost 100%, facilitating ease of research. After seven days of incubation, NS1 could be detected in both orally and intrathoracically infected mosquito lysates via immunoblotting, though in the orally infected mosquitoes the levels of NS1 were much lower (Figure 2A). However, when we tried using the rapid test to detect NS1 in PBS-homogenized orally infected mosquitoes, we observed no positive signals (Figure 2B, upper row). We speculated that our inability to detect NS1 within whole mosquito bodies homogenized with PBS could be the result of undetectable levels or an absence of NS1 secretion.

If the NS1 that accumulated within mosquito cells could be released, NS1 rapid tests would be viable for use for mosquito testing. We therefore investigated whether a detergent-containing buffer (PBS with Triton X-100) could lyse mosquito cells and release NS1, increasing the test’s sensitivity and thus making NS1 testing viable. A single orally infected mosquito was homogenized with Triton X-100-PBS and then tested using a rapid strip test, which resulted in a positive signal (five replicates) (Figure 2B, lower row). These results indicated that the use of detergent to release intracellular NS1 from an orally infected mosquito for NS1 detection is possible, and that the NS1 from a single mosquito was sufficient for rapid test detection.

When an intrathoracically infected mosquito was used to detect NS1 in the same manner, the result was very different from that for the orally infected mosquito. NS1 from mosquitoes infected via thorax injection could be detected in both PBS and Triton X-100-PBS homogenized groups (five replicates) (Figure 2B). NS1 may therefore be present in the hemocoel of mosquitoes infected with DENV via thorax injection. This implies that DENV infection status may differ according to infection route, perhaps due to distinct infected cell types, which may also affect the secretion of NS1.

We used orally infected mosquitoes to determine the concentration of Triton X-100 that was sufficient to lyse mosquito cells. Since the infection rate via oral infection is not 100%, each infected mosquito was bisected longitudinally to provide two samples for testing (Figure 2C); one half was exposed to 0.1%, 0.5% or 1% Triton X-100 in PBS, while the other half was subjected to RT-qPCR testing to confirm the infection status. We found that 0.5% and 1% Triton X-100-PBS allowed intracellular NS1 to be released in sufficient quantities for rapid test detection (Figure 2D).

DENV has four serotypes, and mosquitoes in the field are not limited to serotype 2 infection. The rapid test strip used in this study can detect NS1 from all four serotypes in human samples. Therefore, we infected mosquitoes with each serotype and used them as samples for the rapid test. By using the improved lysis method with the rapid test, NS1 from each serotype could be detected in infected mosquitoes (Figure 2E), indicating that our testing system was not limited to the detection of serotype 2.

### 3.3. NS1 Rapid Test Was Able to Correctly Identify Infected Mosquitoes even during Early Stages of DENV Infection

For the rapid test to be of use in mosquito surveillance, it is important to know when, relative to time of infection, positive tests will be produced. To address this, we screened mosquitoes 3, 5, 7, 14, and 21 days after oral infection individually. Again, each infected mosquito was bisected longitudinally; one half was homogenized with 1% Triton X-100-PBS for use in the NS1 rapid test, while the other was tested using qPCR.

At three days PI, the detection sensitivity using the rapid test was 58.54%. However, by five days PI, this had improved to 89%. This level of sensitivity was maintained through 7, 14 and 21 days PI, with calculated sensitives of 85%, 88% and 87%, respectively (Table 1). Notably, we found no false-positive results in our rapid tests. The calculated Cohen’s Kappa values show moderate agreement at Day 3, Day 7 and Day 21, substantial agreement at Day14 and almost perfect agreement at Day 5. These results, in conjunction with the high sensitivities and specificities seen for each day, suggest that the NS1 rapid test boasts significant potential for use.

### 3.4. Sensitivity of the NS1 Rapid Test Increased after DENV2 Had Spread from the Midgut

**Figure 3:**
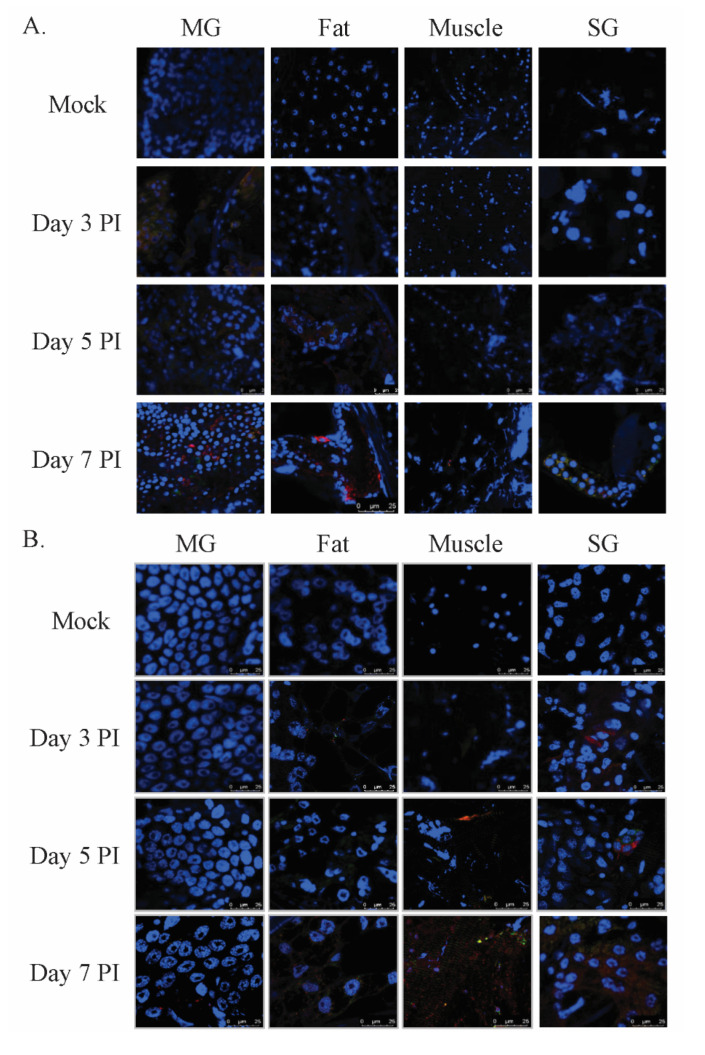
DENV2 progression in infected mosquito bodies. Mosquitoes were infected orally (**A**) or via thorax injection (**B**) with DENV2 and then incubated. At days three, five and seven post-infection (PI), mosquitoes were harvested and dissected to obtain midgut (MG), fat, muscle and salivary glands (SG). All of these tissues were stained with anti-NS1 Ab (red), anti-E Ab (green) and DAPI (blue). The processed tissues were analyzed using confocal microscopy.

To investigate the time from initial infection to the point when DENV-infected mosquitoes were able to transmit the virus, mosquitoes were infected with DENV2 orally and by thorax injection, and were then dissected to harvest midgut, fat tissue, muscle and salivary glands for use in an immunofluorescence assay (Figure 3). Three days post oral infection, the DENV infection was restricted to the midgut tissue, with no obvious infection in other tissues. By five days PI, however, we detected viral protein not only in the midgut, but also in fat tissue, suggesting that the virus was able to cross the midgut barrier [22,23]. By seven days PI, we detected viral NS1 signals in muscles and salivary glands (Figure 3A). In contrast, three days post-infection via intrathoracic injection, we found viral NS1 in fat, muscle and salivary glands (Figure 3B). These results showed that our rapid test was able to stably identify infected mosquitoes after DENV had moved out of the midgut and infected other body tissues. In addition, these results imply that by seven days PI, DENV-infected mosquitoes have the potential to transmit DENV.

## 4. Discussion

**Figure 4:**
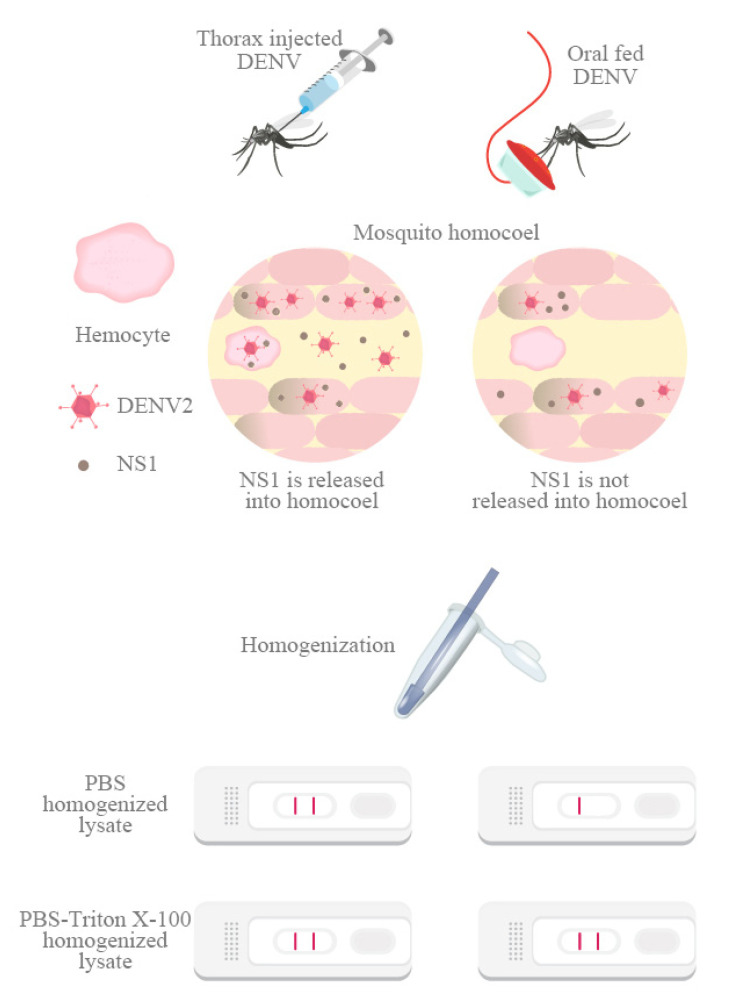
NS1 distribution could be different in intrathoracically and orally infected mosquitoes. Mosquitoes were infected intrathoracically or orally with DENV2. After homogenization, NS1 could be detected in both PBS and Triton X-100-PBS lysates of intrathoracically infected mosquitoes; however, in the orally infected mosquitoes, NS1 could be detected only following Triton X-100-PBS treatment.

The results of this study support the feasibility of the surveillance of mosquito DENV infections by testing for NS1 Ag using a convenient dengue NS1 Ag strip and detergent-containing buffer. We found that in mosquitoes that had been intrathoracically infected with DENV, NS1 could be detected easily without using detergent. This was not the case in orally infected mosquitoes, suggesting that NS1 may be presented in the hemocoel of mosquitoes infected intrathoracically, but not in those infected orally. This implies that NS1 protein levels differ between these two types of infection, potentially because different cell types are infected, and because these different cell types may also secrete different levels of NS1 (Figure 4).

Using our rapid test detection protocol, we individually tested around 40 mosquitoes at designated days after DENV infection. This improved on sample sizes used in previous research [10], and made sensitivity and specificity calculation more reliable. To compare the ability of the NS1 rapid test to detect DENV infection with that of qPCR, we bisected mosquitoes longitudinally (because of left–right symmetry). We found that the sensitivity of the rapid test at three days PI was lower than that at 5–21 days PI, potentially due to the bisection procedure. Our immunofluorescence staining of the mosquito midgut at three days PI showed some infection foci in the midgut tissue (Figure 3A). A previous study also showed a localized infection pattern in the midgut tissue [24]. This suggests that the level of NS1 Ag may vary between the two halves of the mosquito. Small-scale testing using whole mosquitoes as the test sample found a higher sensitivity at three days PI.

In our study, we could not detect NS1 in the culture supernatant of C6/36 cell (Figure 1A) or in PBS-homogenized orally infected mosquito bodies (Figure 2B). Although Tan et al. [10] reported that they could detected NS1 in PBS-homogenized DENV-infected mosquitoes, they used a Bio-Rad Dengue NS1 Ag test strip to detect the presence of NS1. This kit contains a component called migration buffer whose exact content remains unknown, but may contain a material that can damage cell membranes in order to release intracellular NS1. Another previous study reported that NS1 can be detected in the supernatant of mosquito cells by immunoblotting [19], potentially due to the cell line (C6/36HT) and concentrated supernatant. The C6/36HT cell line’s lineage is from C6/36 cells, which can be maintained at higher temperatures (34 °C). In this condition, several flaviviruses demonstrate higher replication rates and viral titers [25], which may result in an increased secretion of NS1. In addition to this, the culture supernatant was further concentrated for NS1 detection, increasing the possibility of NS1 detection. The secretion of NS1 is thus likely limited in mosquito cells, meaning that the detection of NS1 will rely on cell lysis.

The secretion routes of NS1 may also affect the efficiency of NS1 secretion in vertebrates and mosquitoes. In infected mammalian cells, DENV NS1 is secreted via the classic ER–Golgi glycoprotein secretion route [26,27]. In contrast, in infected mosquito cells DENV NS1 secretion seems unrelated to the ER–Golgi glycoprotein secretion route. The NS1 secretion of C6/36HT cells is not significantly inhibited when the *cis* and *trans* cisternae of the Golgi network are disrupted [28,29], or if the assembly of the coat protein II (COPII) complex is blocked, which is required for vesicle transport from the ER to the Golgi [28,29]. The secretion of NS1 by mosquito cells (C6/36HT) may be related to the caveolin-1 (CAV-1)-dependent pathway, which is associated with and relies on the caveolin chaperone complex (CCC) [28,29,30,31]. The efficiency of the CCC-based transport system in terms of NS1 secretion may be limited, because (i) it may not be used only for secretion, or (ii) this secretory route may depend on CCC complex formation. In either case, the secretion of NS1 by the CCC may not be as efficient as the classic ER–Golgi pathway. Regulation of the NS1 secretory pathway may prove to be an important strategy in the prevention of DENV transmission.

In the field, mosquitoes will be captured by traps which may not be emptied daily by researchers. The storage conditions may not be optimal, and hence some mosquitoes may therefore perish before they are collected. Furthermore, DENV infectivity is temperature-related: higher temperatures hasten the spread of DENV within the mosquito [32]. Two studies have reported the successful detection of DENV within infected mosquitoes using NS1 Ag or qPCR after multiple freeze–thaw cycles and/or drying [9,13], even for mosquitoes that had been desiccated for 30 days. To confirm this, we also tested dead infected mosquitoes. Mosquitoes tested either two days or two weeks after death produced clear positive results in both the NS1 rapid test and in qPCR testing. Another study involved screening for DENV in mosquitoes using a different dengue NS1 kit for field-caught mosquitoes [10]. Out of 44 mosquitoes collected in five weeks, two tested positive, suggesting that the NS1 detection system is viable in the field.

Surveillance of virus transmission is an important aspect of preventing DENV outbreaks [33]. The NS1 detection system can provide valuable information for disease control. Detecting NS1 in a mosquito indicates the presence of an infected person in that area at least three days earlier. The proportion of infected to uninfected mosquitoes in an area is likely to be correlated with the number of infected people. This information can be used to identify areas at high risk of a dengue fever outbreak, and disease control plans can be adjusted accordingly. Our modified NS1 detection system could be deployed in the field, offering instant surveillance results and supporting a rapid response from disease control programs. This NS1 detection system could also be used for the validation of mosquito control actions, to ensure the complete elimination of infected mosquitoes after fumigation of an area with insecticides.

## Figures and Tables

**Table 1:** Sensitivity and specificity of the dengue NS1 rapid test at specific days post-infection.

Days Post-Infection	Sample No.	Rapid Test		RT-qPCR		Sensitivity	Specificity	Cohen’s Kappa Agreement
		Positive	Negative	Positive	Negative			
Day 3	41	14	27	24	17	58.33%	100%	Moderate
Day 5	43	24	19	27	16	88.89%	100%	Almost perfect
Day 7	40	25	15	33	7	84.85%	100%	Moderate
Day 14	36	24	12	27	9	88.89%	100%	Substantial
Day 21	36	30	6	34	2	88.24%	100%	Moderate

## Abbreviations

DENV	Dengue virus
NS1	Non-structural protein 1
qPCR	Real-time quantitative polymerase chain reaction
PBS	Phosphate-buffered saline
PI	Post-infection
